# epicontacts: Handling, visualisation and analysis of epidemiological contacts

**DOI:** 10.12688/f1000research.14492.2

**Published:** 2018-10-11

**Authors:** VP Nagraj, Nistara Randhawa, Finlay Campbell, Thomas Crellen, Bertrand Sudre, Thibaut Jombart

**Affiliations:** 1 Research Computing, University of Virginia School of Medicine, Charlottesville, VA, 22903, USA; 2 One Health Institute, University of California, Davis, Davis, CA, 95616, USA; 3 MRC Centre for Outbreak Analysis and Modelling, Department of Infectious Disease Epidemiology, School of Public Health, Imperial College London, London, W2 1PG, UK; 4 Mahidol-Oxford Tropical Medicine Research Unit, Bangkok , 10400, Thailand; 5 European Centre for Disease Prevention and Control, Stockholm, Sweden

**Keywords:** contact tracing, outbreaks, R

## Abstract

Epidemiological outbreak data is often captured in line list and contact format to facilitate contact tracing for outbreak control. *epicontacts *is an R package that provides a unique data structure for combining these data into a single object in order to facilitate more efficient visualisation and analysis. The package incorporates interactive visualisation functionality as well as network analysis techniques. Originally developed as part of the Hackout3 event, it is now developed, maintained and featured as part of the R Epidemics Consortium (RECON). The package is available for download from the Comprehensive R Archive Network (CRAN) and GitHub.

## Introduction

In order to study, prepare for, and intervene against disease outbreaks, infectious disease modellers and public health professionals need an extensive data analysis toolbox. Disease outbreak analytics involve a wide range of tasks that need to be linked together, from data collection and curation to exploratory analyses, and more advanced modelling techniques used for incidence forecasting ^1, 2^ or to predict the impact of specific interventions ^3, 4^. Recent outbreak responses suggest that for such analyses to be as informative as possible, they need to rely on a wealth of available data, including timing of symptoms, characterisation of key delay distributions (e.g. incubation period, serial interval), and data on contacts between patients ^5– 8^.

The latter type of data is particularly important for outbreak analysis, not only because contacts between patients are useful for unravelling the drivers of an epidemic ^9, 10^, but also because identifying new cases early can reduce ongoing transmission via contact tracing, i.e. follow-up of individuals who reported contacts with known cases ^11, 12^. However, curating contact data and linking them to existing line lists of cases is often challenging, and tools for storing, handling, and visualising contact data are often missing ^13, 14^.

Here, we introduce epicontacts, an R ^15^ package providing a suite of tools aimed at merging line lists and contact data, and providing basic functionality for handling, visualising and analysing epidemiological contact data. Maintained as part of the R Epidemics Consortium ( RECON), the package is integrated into an ecosystem of tools for outbreak response using the R language.

## Use cases

Those interested in using epicontacts should have a line list of cases as well as a record of contacts between individuals. Both datasets must be enumerated in tabular format with rows and columns. At minimum the line list requires one column with a unique identifier for every case. The contact list needs two columns for the source and destination of each pair of contacts. The datasets can include arbitrary features of case or contact beyond these columns. Once loaded into R and stored as data.frame objects, these datasets can be passed to the make_epicontacts() function (see ‘Methods’ section for more detail). For an example of data prepared in this format, users can refer to the outbreaks R package.

# load the outbreaks package
library(outbreaks)

# example simulated ebola data

# line list
str(ebola_sim$linelist)

## ‘data.frame’:    5888 obs. of 9 variables:
##  $ case_id                : chr "d1fafd" "53371b" "f5c3d8" "6c286a" ...
##  $ generation             : int 0 1 1 2 2 0 3 3 2 3 ...
##  $ date_of_infection      : Date, format: NA "2014-04-09" ...
##  $ date_of_onset          : Date, format: "2014-04-07" "2014-04-15" ...
##  $ date_of_hospitalisation: Date, format: "2014-04-17" "2014-04-20" ...
##  $ date_of_outcome        : Date, format: "2014-04-19" NA ...
##  $ outcome                : Factor w/ 2 levels "Death","Recover": NA NA 2 1 2 NA 2 1 2 1 ...
##  $ gender                 : Factor w/ 2 levels "f","m": 1 2 1 1 1 1 1 1 2 2 ...
##  $ hospital               : Factor w/ 11 levels "Connaught Hopital",..: 4 2 7 NA 7 NA 2 9 7 11 ...

# contact list
str(ebola_sim$contacts)

## ’data.frame’:    3800 obs. of  3 variables:
##  $ infector: chr  "d1fafd" "cac51e" "f5c3d8" "0f58c4" ...
##  $ case_id : chr  "53371b" "f5c3d8" "0f58c4" "881bd4" ...
##  $ source  : Factor w/ 2 levels "funeral","other": 2 1 2 2 2 1 2 2 2 2 ...

# example middle east respiratory syndrome data

# line list
str(mers_korea_2015$linelist)

## ’data.frame’:    162 obs. of 15 variables:
##  $ id            : chr "SK_1" "SK_2" "SK_3" "SK_4" ...
##  $ age           : int 68 63 76 46 50 71 28 46 56 44 ...
##  $ age_class     : chr "60-69" "60-69" "70-79" "40-49" ...
##  $ sex           : Factor w/ 2 levels "F","M": 2 1 2 1 2 2 1 1 2 2 ...
##  $ place_infect  : Factor w/ 2 levels "Middle East",..: 1 2 2 2 2 2 2 2 2 2 ...
##  $ reporting_ctry: Factor w/ 2 levels "China","South Korea": 2 2 2 2 2 2 2 2 2 1 ...
##  $ loc_hosp      : Factor w/ 13 levels "365 Yeollin Clinic, Seoul",..: 10 10 10 10 1 10 10 13 10 10 ...
##  $ dt_onset      : Date, format: "2015-05-11" "2015-05-18" ...
##  $ dt_report     : Date, format: "2015-05-19" "2015-05-20" ...
##  $ week_report   : Factor w/ 5 levels "2015_21","2015_22",..: 1 1 1 2 2 2 2 2 2 2 ...
##  $ dt_start_exp  : Date, format: "2015-04-18" "2015-05-15" ...
##  $ dt_end_exp    : Date, format: "2015-05-04" "2015-05-20" ...
##  $ dt_diag       : Date, format: "2015-05-20" "2015-05-20" ...
##  $ outcome       : Factor w/ 2 levels "Alive","Dead": 1 1 2 1 1 2 1 1 1 1 ...
##  $ dt_death      : Date, format: NA NA ...

# contact list
str(mers_korea_2015$contacts)

## ’data.frame’:    98 obs. of  4 variables:
##  $ from         : chr  "SK_14" "SK_14" "SK_14" "SK_14" ...
##  $ to           : chr  "SK_113" "SK_116" "SK_41" "SK_112" ...
##  $ exposure     : Factor w/ 5 levels "Contact with HCW",..: 2 2 2 2 2 2 2 2 2 2 ...
##  $ diff_dt_onset: int  10 13 14 14 15 15 15 16 16 16 ...

The data handling, visualization, and analysis methods described above represent the bulk of epicontacts features. More examples of how the package can be used as well as demonstrations of additional features can be found through the RECON learn platform and the epicontacts vignettes.

## Methods

### Operation

epicontacts is released as an open-source R package. A stable release is available for Windows, Mac and Linux operating systems via the CRAN repository. The latest development version of the package is available through the RECON Github organization. At minimum users must have R installed. No other system dependencies are required.

# install from CRAN
install.packages("epicontacts")

# install from Github
install.packages("devtools")
devtools::install_github("reconhub/epicontacts")

# load and attach the package
library(epicontacts)

### Implementation

***Data handling.*** epicontacts includes a novel data structure to accommodate line list and contact list datasets in a single object. This object is constructed with the make_epiconctacts() function and includes attributes from the original datasets. Once combined, these are mapped internally in a graph paradigm as nodes and edges. The epicontacts data structure also includes a logical attribute for whether or not this resulting network is directed.

The package takes advantage of R’s generic functions, which call specific methods depending on the class of an object. This is implemented several places, including the summary.epicontacts() and print.epicontacts() methods, both of which are respectively called when the summary() or print() functions are used on an epicontacts object. The package does not include built-in data, as exemplary contact and line list datasets are available in the outbreaks package ^16^.

The example that follows will use the mers_korea_2015 dataset from outbreaks, which which includes initial data collected by the Epidemic Intelligence group at European Centre for Disease Prevention and Control (ECDC) during the 2015 outbreak of Middle East respiratory syndrome (MERS-CoV) in South Korea. Note that the data used here was provided in outbreaks for teaching purposes, and therefore does not include the complete line list or contacts from the outbreak.

# install the outbreaks package for data
install.packages("outbreaks")

# load the outbreaks package
library(outbreaks)

# construct an epicontacts object
x <- make_epicontacts(linelist=mers_korea_2015[[1]],
                         contacts = mers_korea_2015[[2]],
                         directed=TRUE)

# print the object
x



## 
## /// Epidemiological Contacts /// 
## 
## // class: epicontacts 
## // 162 cases in linelist; 98 contacts;  directed 
## 
## // linelist 
## 
## ’data.frame’:    162 obs. of 15 variables:
##  $ id            : chr "SK_1" "SK_2" "SK_3" "SK_4" ...
##  $ age           : int 68 63 76 46 50 71 28 46 56 44 ...
##  $ age_class     : chr "60-69" "60-69" "70-79" "40-49" ...
##  $ sex           : Factor w/ 2 levels "F","M": 2 1 2 1 2 2 1 1 2 2 ...
##  $ place_infect  : Factor w/ 2 levels "Middle East",..: 1 2 2 2 2 2 2 2 2 2 ...
##  $ reporting_ctry: Factor w/ 2 levels "China","South Korea": 2 2 2 2 2 2 2 2 2 1 ...
##  $ loc_hosp      : Factor w/ 13 levels "365 Yeollin Clinic, Seoul",..: 10 10 10 10 1 10 10 13 10 10 ...
##  $ dt_onset      : Date, format: "2015-05-11" "2015-05-18" ...
##  $ dt_report     : Date, format: "2015-05-19" "2015-05-20" ...
##  $ week_report   : Factor w/ 5 levels "2015_21","2015_22",..: 1 1 1 2 2 2 2 2 2 2 ...
##  $ dt_start_exp  : Date, format: "2015-04-18" "2015-05-15" ...
##  $ dt_end_exp    : Date, format: "2015-05-04" "2015-05-20" ...
##  $ dt_diag       : Date, format: "2015-05-20" "2015-05-20" ...
##  $ outcome       : Factor w/ 2 levels "Alive","Dead": 1 1 2 1 1 2 1 1 1 1 ...
##  $ dt_death      : Date, format: NA NA ...
##
## // contacts
##
## ’data.frame’:    98 obs. of  4 variables:
##  $ from         : chr  "SK_14" "SK_14" "SK_14" "SK_14" ...
##  $ to           : chr  "SK_113" "SK_116" "SK_41" "SK_112" ...
##  $ exposure     : Factor w/ 5 levels "Contact with HCW",..: 2 2 2 2 2 2 2 2 2 2 ...
##  $ diff_dt_onset: int  10 13 14 14 15 15 15 16 16 16 ...

# view a summary of the object 
summary(x)


##
## /// Overview //
##   // number of unique IDs in linelist: 162
##   // number of unique IDs in contacts: 97
##   // number of unique IDs in both: 97
##   // number of contacts: 98
##   // contacts with both cases in linelist: 100 %
##
## /// Degrees of the network //
##   // in-degree summary:
##    Min. 1st Qu.  Median    Mean 3rd Qu.    Max.
##    0.00    1.00    1.00    1.01    1.00    3.00
##
##   // out-degree summary:
##    Min. 1st Qu.  Median    Mean 3rd Qu.    Max.
##    0.00    0.00    0.00    1.01    0.00   38.00
##
##   // in and out degree summary:
##    Min. 1st Qu.  Median    Mean 3rd Qu.    Max.
##   1.000   1.000   1.000   2.021   1.000  39.000
##
## /// Attributes //
##   // attributes in linelist:
##  age age_class sex place_infect reporting_ctry loc_hosp dt_onset dt_report week_report dt_start_exp dt_end_exp dt_diag outcome dt_death
##
##   // attributes in contacts:
##  exposure diff_dt_onset

**Figure 1.**
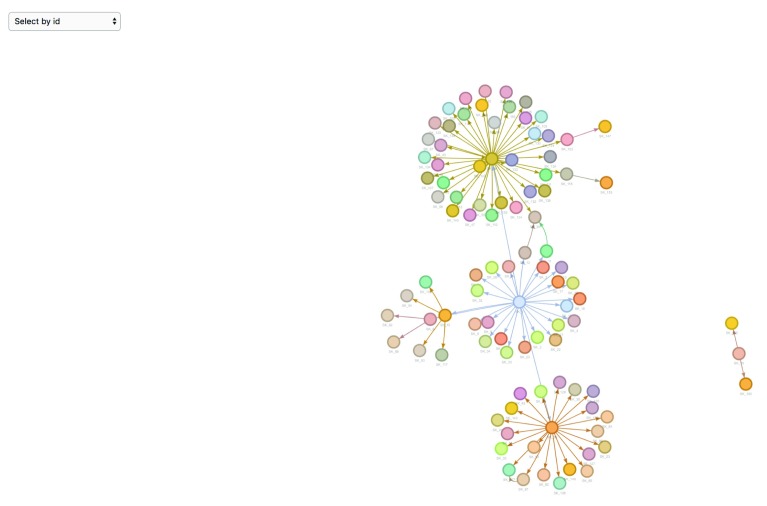
The generic plot() method for an epicontacts object will use the visNetwork method by default.

**Figure 2.**
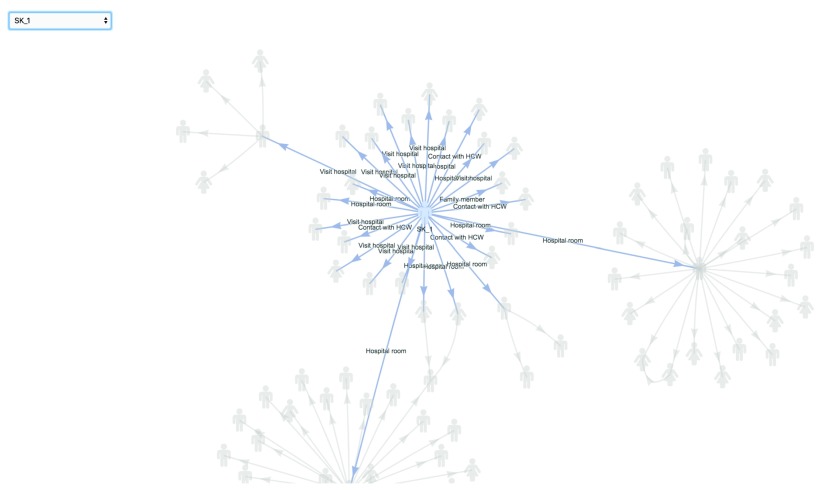
The vis_epicontacts() function explicitly calls visNetwork to make an interactive plot of the contact network.

**Figure 3.**
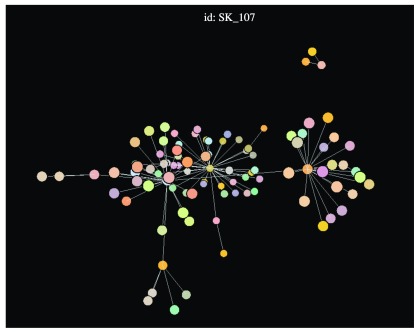
The graph3D() function generates a three-dimensional network plot.

***Data visualisation.*** epicontacts implements two interactive network visualisation packages: visNetwork and threejs ^17, 18^. These frameworks provide R interfaces to the vis.js and three.js JavaScript libraries respectively. Their functionality is incorporated in the generic plot() method ( Figure 1) for an epicontacts object, which can be toggled between either with the “type” parameter. Alternatively, the visNetwork interactivity is accessible via vis_epicontacts() ( Figure 2), and threejs through graph3D() ( Figure 3). Each function has a series of arguments that can also be passed through plot(). Both share a color palette, and users can specify node, edge and background colors. However, vis_epicontacts() includes a specification for “node_shape” by a line list attribute as well as a customization of that shape with an icon from the Font Awesome icon library. The principal distinction between the two is that graph3D() is a three-dimensional visualisation, allowing users to rotate clusters of nodes to better inspect their relationships.

plot(x)

vis_epicontacts(x,
		  node_shape = "sex",
		  shapes = c(F = "female", M = "male"),
		  edge_label = "exposure")

graph3D(x, bg_col = "black")

***Data analysis.*** Subsetting is a typical preliminary step in data analysis. epicontacts leverages a customized subset method to filter line lists or contacts based on values of particular attributes from nodes, edges or both. If users are interested in returning only contacts that appear in the line list (or vice versa), the thin() function implements such logic.

# subset for males
subset(x, node_attribute = list("sex" = "M"))

# subset for exposure in emergency room
subset(x, edge_attribute = list("exposure" = "Emergency room"))

# subset for males who survived and were exposed in emergency room
subset(x,
        node_attribute = list("sex" = "M", "outcome" = "Alive"),
        edge_attribute = list("exposure" = "Emergency room"))

thin(x, "contacts")
thin(x, "linelist")

For analysis of pairwise contact between individuals, the get_pairwise() feature searches the line list based on the specified attribute. If the given column is a numeric or date object, the function will return a vector containing the difference of the values of the corresponding “from” and “to” contacts. This can be particularly useful, for example, if the line list includes the date of onset of each case. The subtracted value of the contacts would approximate the serial interval for the outbreak ^19^. For factors, character vectors and other non-numeric attributes, the default behavior is to print the associated line list attribute for each pair of contacts. The function includes a further parameter to pass an arbitrary function to process the specified attributes. In the case of a character vector, this can be helpful for tabulating information about different contact pairings with table().

# find interval between date onset in cases
get_pairwise(x, "dt_onset")

# find pairs of age category contacts
get_pairwise(x, "age_class")

# tabulate the pairs of age category contacts
get_pairwise(x, "age_class", f = table)

## Discussion

### Benefits

While there are software packages available for epidemiological contact visualisation and analysis, none aim to accommodate line list and contact data as purposively as epicontacts ^20– 22^. Furthermore, this package strives to solve a problem of plotting dense graphs by implementing interactive network visualisation tools. A static plot of a network with many nodes and edges may be difficult to interpret. However, by rotating or hovering over an epicontacts visualisation, a user may better understand the data.

### Future considerations

The maintainers of epicontacts anticipate new features and functionality. Future development could involve performance optimization for visualising large networks, as generating these interactive plots is resource intensive. Additionally, attention may be directed towards inclusion of alternative visualisation methods.

## Conclusions

epicontacts provides a unified interface for processing, visualising and analyzing disease outbreak data in the R language. The package and its source are freely available on CRAN and GitHub. By developing functionality with line list and contact list data in mind, the authors aim to enable more efficient epidemiological outbreak analyses.

## Software availability

Software available from: https://CRAN.R-project.org/package=epicontacts

Source code available from: https://github.com/reconhub/epicontacts

Archived source code as at time of publication: https://zenodo.org/record/1210993 ^23^

Software license: GPL 2
