# Supplementary material for: epicontacts: Handling, visualisation and analysis of epidemiological contacts
Source: F1000Res. 2018 Oct 11;7:566. Originally published 2018 May 10. [Version 2] doi: 10.12688/f1000research.14492.2 (PMC6572866; doi:10.12688/f1000research.14492.2)
Supplement: Peer review history

# Peer review: Reviewer response for version 2

**DOI:** 10.5256/f1000research.18105.r49529

**Published:** 2019-06-14

**Authors:** Josie Athens

**Affiliations:** 1 Department of Preventive and Social Medicine, University of Otago, Dunedin, New Zealand

The authors present an R package that helps in the visualisation and analysis of epidemiological contacts.

 It is a well-presented summary of the capabilities of the software and brings network theory tools to academics from the areas of public health and epidemiology.

 For version 2 of the manuscript, the authors follow the Introduction with Use cases, which benefits the understanding of how data has to be prepared for input. I followed the examples, and the output that they present about the ebola data set is outdated. I suggest to include the version of the outbreaks package used in the examples. For those not familiar with R, presenting the data with the str command is not clear; to use the command head instead, could be a better option. This is something minor and not needed but would improve the understanding of the software on the most critical point, which is data input.

 As in version 2, the Use cases section is presented before the Methods section, the last paragraph from the Use cases section needs to be changed from: "methods described above" to "methods described below".

 The visualisations produced from epicontacts are impressive. For the manuscript to become more accessible to main stream readers, it would need to include the output from the analysis and interpretation.

I have read this submission. I believe that I have an appropriate level of expertise to confirm that it is of an acceptable scientific standard.

# Peer review: Reviewer response for version 1

**DOI:** 10.5256/f1000research.15777.r36044

**Published:** 2018-08-02

**Authors:** Peter Adebayo Adewuyi

**Affiliations:** 1 Liberia Field Epidemiology Training Program, Monrovia, Liberia; 2 African Field Epidemiology Network (AFENET), Kampala, Uganda

This is a good software developed which could help in continuous visualization of contacts and their progression in disease tracking. 

 It is user friendly for those who are not computer specialist and still want to visualize data.

 Data visualization is pertinent to disease monitoring and what the authors have done will aid in helping epidemiologist and public health specialist involved in outbreak response to quickly visualize progression and spread of disease from primary to secondary contacts and how the disease is evolving among contacts.

 The software will actually achieve its purpose as stated in the conclusion of the write-up. Good work done.

I have read this submission. I believe that I have an appropriate level of expertise to confirm that it is of an acceptable scientific standard.

# Peer review: Reviewer response for version 1

**DOI:** 10.5256/f1000research.15777.r34084

**Published:** 2018-05-31

**Authors:** Melissa A. Rolfes

**Affiliations:** 1 Centers for Disease Control and Prevention (CDC) , Atlanta, GA, USA

The article describes an R-based software tool aimed to facilitate analysis of data from outbreaks that include line lists of cases and case-contact data. The R package, epicontacts, is part of a larger suite of tools housed at the R Epidemics Consortium (RECON). The epicontacts package has the ability to merge data about cases in a line list with case-contact details, which then allows the user to describe and visualize contact networks, incubation periods, and serial intervals within an outbreak.

 The codes and methods for analysis are partly described in the article, and the authors should provide a link to the packages documentation, either at CRAN or RECON webpages, where readers could learn more about the package and its options.

 The output of the package provided in the article was interesting and intriguing. I felt that it was only partly explained and the article could benefit from the authors annotating the output and its interpretation a bit further. I have explored the RECON website and found the RECON Learn modules to be quite helpful in providing annotation of the epicontacts output and some guidance on interpretation. I would recommend that the authors consider either expanding the annotation of the output in this article or explicitly direct readers to the RECON Learn website for further instruction. 

 Additional suggestions:

- Consider moving the section of the article called "Use cases" to before the "Data handling" subsection of the "Implementation" section. I felt that the description of the input datasets under "Use cases" was very informative and would have been organizational more helpful had it been placed earlier in the article.
-  Consider describing the sample outbreak data in a bit further detail. It appears to be data describing the MERS outbreak that occurred in South Korea in 2015. I think the description should include whether the data are simulated or from a real outbreak (if from a real outbreak, then a reference to the outbreak description should be included), the scenario of the outbreak, how many cases, how many contacts, place of the outbreak, duration of the outbreak, and a brief description of the demographic details included in the dataset. This amount of detail would allow the reader to translate the details of the outbreak from your text to the output provided by epicontacts.

I have read this submission. I believe that I have an appropriate level of expertise to confirm that it is of an acceptable scientific standard, however I have significant reservations, as outlined above.
